# Key considerations in the adoption of Artificial Intelligence in public health

**DOI:** 10.1371/journal.pdig.0000540

**Published:** 2024-07-01

**Authors:** Itai Bavli, Sandro Galea

**Affiliations:** 1 School of Population and Public Health, The University of British Columbia, Vancouver, British Columbia, Canada; 2 School of Public Health, Boston University, Boston, Massachusetts, United States of America; Mayo Clinic Arizona, UNITED STATES

## Abstract

The integration of Artificial Intelligence (AI) into public health has the potential to transform the field, influencing healthcare at the population level. AI can aid in disease surveillance, diagnosis, and treatment decisions, impacting how healthcare professionals deliver care. However, it raises critical questions about inputs, values, and biases that must be addressed to ensure its effectiveness. This article investigates the factors influencing the values guiding AI technology and the potential consequences for public health. It outlines four key considerations that should shape discussions regarding the role of AI in the future of public health. These include the potential omission of vital factors due to incomplete data inputs, the challenge of balancing trade-offs in public health decisions, managing conflicting inputs between public health objectives and community preferences, and the importance of acknowledging the values and biases embedded in AI systems, which could influence public health policy-making.

Artificial intelligence (AI) is currently attracting significant attention in both the scientific literature and the popular media. These discussions have started to explore the potential impact of deploying this technology in medicine [1,2], highlighting both the potential of AI, and its potential challenges. While there is no question that AI can have immediate implications for clinical medicine, it is not difficult to imagine that AI can also quickly become an important tool in the arsenal of public health [3,4]. The work of public health involves both foundational work of surveillance and mitigation of disease spread (i.e., protective interventions), and the long-term work of changing environments to the end of creating contexts that build health [5]. This work all involves computational population health science thinking, and the assessment of trade-offs needed to invest in particular efforts to generate health. AI’s ability to help synthesize available information efficiently can lend itself well to the work of public health.

Anticipating such emergence of AI in short order, it seems pertinent to question some of the foundational assumptions that underlie the work of AI, and that may influence AI’s utility for public health. Central to these are the questions of inputs, values, and biases. For example, a recent analysis examined the ideological orientation of OpenAI ChatGPT, revealing a pro-environmental, left-libertarian ideology [6]. Generative AI’s ideology can stem from the data the system is trained upon, feedback from human users who manually train the system (e.g., ranking prompt solutions), and the inherent filters embedded in the system aimed at preventing the dissemination of harmful content [6]. The existence of these biases could also potentially be attributed to the values held by its creators, leading to a key question: what is informing the values that inform AI technology, and what implications may this have for public health? We outline here four key considerations that should shape conversations about the future of AI in public health (Fig 1).

**Fig 1:**
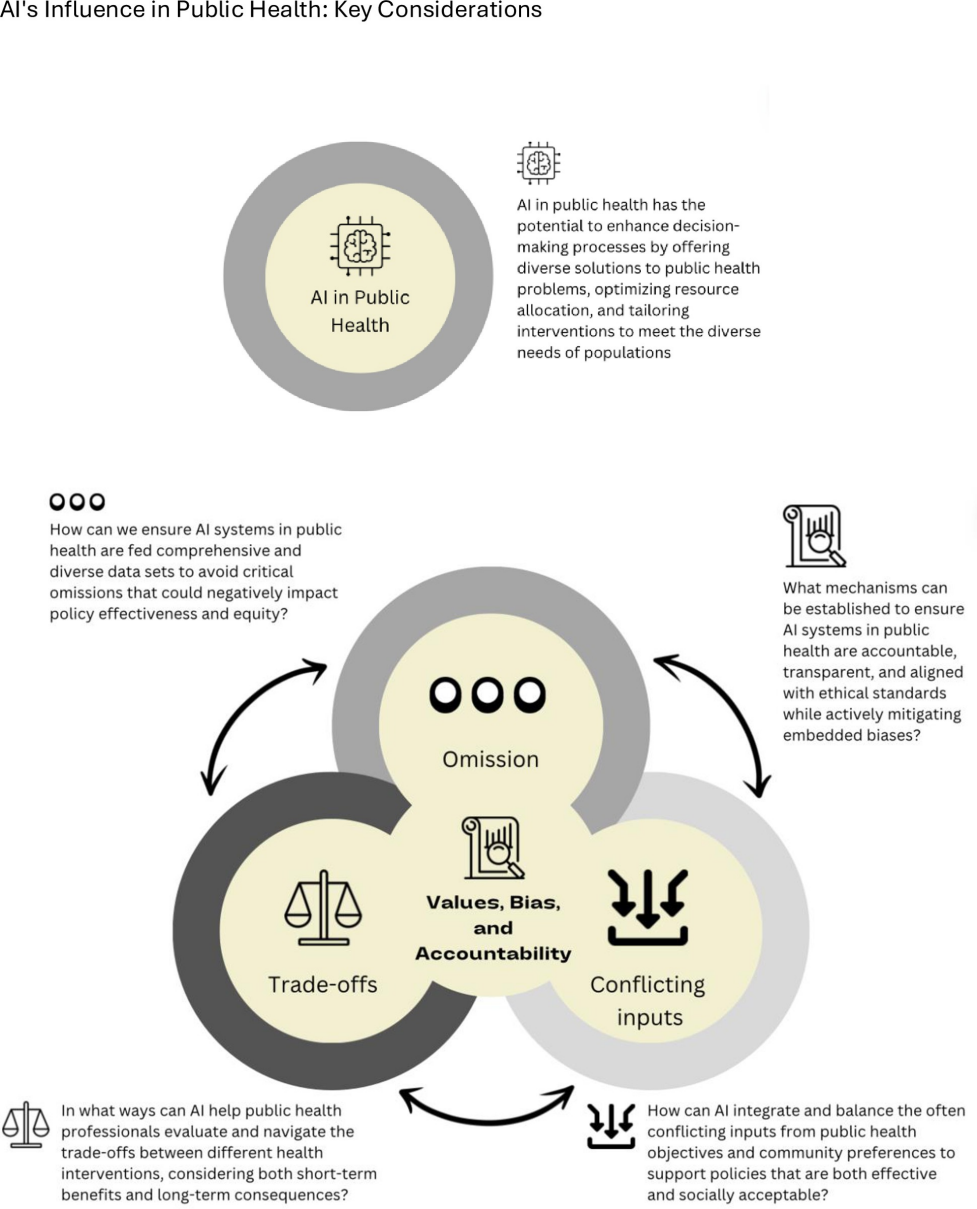
AI’s influence in public health: Key considerations.

## Omission

Generative AI relies on the data fed into the system to generate outcomes aimed at satisfying users. However, these data can often be incomplete and limited. Consequently, the scope of solutions they offer for addressing a particular public health issue may be constrained by the data currently available to the system. This limitation can result in the omission of critical factors influencing our health. For instance, consider the influence of commercial factors on our well-being, such as the strategies employed by the private sector to promote products that can be detrimental to health [7]. Neglecting these considerations in public health may lead to policies that fail to address and respond to significant actions by private actors (e.g., the tobacco and food industries) that affect the heath of the public. Consider a public health campaign aimed at reducing vaping among teenagers that does not take into account the marketing tactics used by manufacturers. While not unique to AI, but possibly worsened by AI’s systematic omissions, the question of what is left out of the analysis and what AI fails to consider when assisting public health thus becomes crucial.

## Trade-offs

Essential to public health is the capacity to assess a given problem from multiple perspectives, considering potential trade-offs such as the costs and benefits of a particular intervention [8]. In theory, generative AI presents itself as a valuable tool for contemplating diverse perspectives simply by generating different solutions. However, it remains uncertain how AI conducts cost-benefit analyses and assigns weight to various alternatives. For example, if the algorithm is designed to maximize health benefits, it may disregard community perspectives. Consider, for example, speed limits. How will AI determine whether we should maintain a universal speed limit of 25 mph? Data amply show that doing so would reduce mortality nationwide [9]. Will AI therefore incorporate the values of expedience and community wish for more rapid transit into the analysis or exclude it entirely?

## Conflicting inputs

This brings us to a related challenge that can emerge from the integration of AI in public health: the presence of conflicting inputs between public health objectives and community preferences. This issue is not unique to AI but is rather a recurring challenge in public health that has, thus far, been addressed through human discussion and debate. Public health often adopts a paternalistic approach [10], dictating what is beneficial for individuals’ health, sometimes against their explicit wishes. The paternalistic nature of public health frequently generates tension between the public health profession, which aims to safeguard individuals’ lives, and the needs and concerns of communities. In such conflicts, solutions must emerge through engaging communities and through finding ways to incorporate such concerns into the decision-making process. It is unclear if AI can incorporate such conflicting inputs, and how the perspectives can be suitably weighted to reach the best solution for many.

In its current form, AI is incapable of engaging with communities and incorporating their inputs into the decision-making process in the same manner as public health professionals. Although community voices can be discovered online and referenced by AI, the process of community engagement is complex. Consider, for example, the scenario of community-based clinics that allow childbirth within the community. This approach may be at conflict with the goal of ensuring the safety of mothers and their infants, particularly in the absence of trained healthcare providers for optimal care, especially during emergencies and in resource-constrained settings. It is unlikely that AI will capture this complexity and comprehend calls for the de-medicalization of childbirth without actively involving communities and attentively listening to their needs. Consequently, this situation may contribute to, or exacerbate, unnecessary tensions between public health experts and communities.

## Values, bias, and accountability

Recent writing has emphasized that the use of Large Language Models (LLMs) in medicine needs to be guided and shaped by the medical profession—ensuring the verification of its values—and not by technology companies or other sources [11]. These discussions are also important for public health. AI does not operate in an objective, value-free realm. Whether values are already embedded in the system [6] or created and amplified by users, these values and the biases they create may impact public health policies.

It is hard to predict how AI embedded values will influence public health. Could the adoption of AI lead to a shift in priorities within public health, such as placing greater emphasis on mitigating disease spread while diminishing attention to addressing the social determinants of health and health equity? How might this affect communities that regularly encounter public health policies grounded in values significantly different from their own? Could it potentially strain relations with the medical profession and erode the trust these populations have in those responsible for making public health decisions?

Along with these questions, comes the issue of accountability: who should be held accountable for AI informed public health policies that fail to protect the public or, in rare instances, harm the public? Failure can occur at different stages of the policy process [12], and it is unclear how AI will affect the accountability chain (e.g., decision-making, implementation, or evaluation processes). This also raises the issue of liability. Frameworks for liability issues (e.g., malpractice) in the integration of AI in clinical care suggest a broader focus on the different stakeholders, beyond clinicians, who are liable for algorithmic inaccuracies that can harm patients [13]. While public health is different from clinical practice (it is rare for public health policies to be sued for failure to protect or harm the public), questions of accountability and liability are important and must not be ignored.

## Conclusion

It is reasonable to anticipate that AI will play a meaningful role in public health, assisting public health professionals in safeguarding the well-being of communities. Consequently, it becomes imperative to prioritize transparency concerning the inputs, values, and biases that guide these systems and the potential challenges associated with integrating AI into public health.
